# Association of Nature‐Based (Satoyama) Activities and Multiple Health Outcomes Among Older Adults: A Pilot Study

**DOI:** 10.1111/ggi.70358

**Published:** 2026-01-20

**Authors:** Weida Lyu, Tomoki Tanaka, Masaharu Koshizuka, Tsutomu Katagiri, Tatsuzou Hamada, Koichirou Kani, Kensaku Nishihara, Yuriko Yamamoto, Kensuke Fukushi, Katsuya Iijima

**Affiliations:** ^1^ Institute of Gerontology, the University of Tokyo Tokyo Japan; ^2^ Institute for Future Initiatives, the University of Tokyo Tokyo Japan; ^3^ Forestry and Nature Engagement Division, Department of Environmental and Industrial Affairs Hadano Kanagawa Japan; ^4^ NPO Corporation Nature School Tanzawa Don‐Kai Hadano Kanagawa Japan; ^5^ AEON Environmental Foundation Tokyo Japan

Dear Editor,

1

“Satoyama” refers to traditional socio‐ecological landscapes in rural Japan, shaped by the harmonious interaction between humans and nature, typically consisting of woodlands, farmlands, grasslands, and ponds near settlements. Satoyama areas account for about 39.4% of Japan's land and are distributed nationwide, reflecting diverse land‐use patterns. They tend to appear in transitional zones between urban districts and surrounding forests, especially in suburban regions. While they are limited in some mountainous areas with continuous forests, Satoyama landscapes remain common around many major metropolitan areas. These peri‐urban Satoyama zones function as important ecological buffers and provide accessible natural environments for nearby residents [1].

While valued for their ecological and cultural significance, these landscapes are increasingly threatened by depopulation, land abandonment, environmental degradation, and the erosion of traditional knowledge due to rural aging and urbanization. The representative photographs of a Satoyama landscape are shown in Supplementary Figure 1. As part of an effort to revitalize these activities and promote intergenerational participation, we engaged in a broader initiative combining research and social action. Beyond environmental value, Satoyama activities appear to benefit older adults' physical and mental health. Nature‐based interventions have been shown to promote physical, psychological, and social health among older adults [2]. Participation in Satoyama activities may also improve psychophysiological well‐being across generations, particularly in post‐pandemic communities [3]. Moreover, as traditional socio‐ecological systems, Satoyama landscapes contribute to human well‐being by supporting cultural identity, local livelihoods, and intergenerational exchange, alongside their environmental value [4]. However, empirical evidence linking Satoyama activities to multidimensional health outcomes remains limited. This pilot study examined associations between Satoyama activities and physical, oral, psychosocial health among community‐dwelling older adults.

In this study, we enrolled 21 older adults who regularly participated in Satoyama activities in Hadano City, a mid‐sized city in western Kanagawa Prefecture Japan, with a population of about 159 910 in 2025. It is located roughly 60–70 km southwest of central Tokyo. The city sits between the Tanzawa Mountains in the north and the Shibusawa Hills in the south, with the Hadano Basin‐Kanagawa's only typical fault‐block basin lying in the middle. Hadano City used to be a major leaf‐tobacco farming area, and Satoyama forests played an important role by providing fertilizer and fuel. After tobacco farming declined, many Satoyama areas were no longer used. Today, however, local community groups and the city government are working together to restore and protect these landscapes. After excluding those with short‐term participation (< 3 months), low frequency (< 1/week), and age < 65, 19 participants remained. Control group was the community‐dwelling older adults above 65 years old who participated the 2024 Kashiwa Cohort Study follow‐up, a prospective cohort study conducted in Kashiwa city, Chiba Prefecture in Japan, using the same inclusion criteria [5]. The study was approved by the University of Tokyo Ethics Committee (No. 24‐239), with anonymized data.

To address confounding, we performed 1:2 nearest neighbor propensity score matching (PSM, caliper = 0.2) without replacement, using age, sex, BMI, and living arrangement as covariates [6]. PSM was chosen to reduce selection bias and better isolate the effect of Satoyama activities. The final sample included 14 Satoyama participants and 28 matched non‐participants. To evaluate the discriminative ability of the propensity score model, we calculated the area under the receiver operating characteristic (ROC) curve (AUC). The resulting AUC was 0.815, indicating good discriminatory power in distinguishing between Satoyama activity participants and non‐participants. Frailty was further assessed using the Cardiovascular Health Study (CHS) criteria, the score ranges from 0 to 5, as described in our previous study [5]. In this study, a CHS score of 0 indicated non‐frailty, and scores ≥ 1 were classified as frailty.

Baseline characteristics were comparable between groups (Table 1), with no significant differences in age, sex, BMI, grip strength, TUG, ASMI, oral function, oral health (General Oral Health Assessment lndex, GOHAI) [7], or social support [8]. However, the Satoyama group showed significantly faster gait speed, lower frailty prevalence, and marginally higher social network scores (Lubben social network) [9]. Furthermore, univariate analysis (Table S1) showed that Satoyama activities were significantly associated with faster gait speed and greater social networks. The frailty score was marginally lower, while no significant differences were found in grip strength, TUG, ASMI, oral motor skill, or GOHAI. Furthermore, Firth logistic regression was applied due to the small sample size and quasi‐separation. Satoyama activities were significantly associated with a lower likelihood of frailty (Table S2).

**TABLE 1:** Participant characteristics according to the Satoyama activities status.

	Overall		Satoyama activities				*p* *
			No		Yes		
No. of participants, *n*	*n* = 42		*n* = 28		*n* = 14		—
Basic characteristics							
Age, years	75.1	±3.7	75.3	±3.9	74.8	±3.4	0.607^a^
Sex, women, *n* (%)	21	50.0%	15	53.6%	6	42.9%	0.513^b^
Living alone, *n* (%)	3	7.1%	2	7.1%	1	7.1%	1.00^b^
Physical function							
Body mass index, kg/m^2^	21.2	±3.6	20.9	±2.9	21.9	±4.7	0.405^a^
ASMI, kg/m^2^	6.6	±1.0	6.4	±0.9	6.9	±1.2	0.228^a^
Grip strength, kg	28.0	±7.3	27.2	±6.9	29.6	±8.0	0.284^a^
Gait speed, m/s	1.4	±0.2	1.4	±0.2	1.5	±0.2	0.020^a^
Timed Up and Go test, s	5.4	±0.6	5.5	±0.7	5.3	±0.5	0.436^a^
Oral function							
Articulatory oral motor skill, “ka,” times/s	6.0	±0.7	6.2	±0.7	5.8	±0.8	0.101^a^
GOHAI	53.6	±5.5	53.1	±5.5	54.4	±5.6	0.405^a^
Sociality							
Social network, lsns6	26.5	±5.3	25.4	±5.9	28.8	±2.6	0.080^a^
Social support	3.6	±0.7	3.5	±0.7	3.8	±0.6	0.284^a^
Frailty status, yes, *n* (%)	25	59.5%	20	71.4%	5	35.7%	0.026^b^

*Note:* Frailty status, assessed using the Cardiovascular Health Study criteria, the score ranges from 0 to 5. 0, non‐frailty; ≥ 1, frailty.

Abbreviations: ASMI, Appendicular Skeletal Muscle Mass Index; GOHAI, General Oral Health Assessment Index.

* The differences in participant characteristics were analyzed using the following: ^a^Unpaired *t* test; ^b^Pearson Chi‐square test.

This study found that Satoyama activities may be associated with faster walking speed, wider social networks, and lower prevalence of frailty in older adults. Previous studies have also shown that activities such as walking on uneven terrain and engaging in light physical labor may help improve lower limb strength [10]. Previous studies have linked nature‐based interventions, such as gardening and forestry programs, to improvements in physical, mental, and social health [1, 2]. Other studies have also shown potential benefits of these interventions on mood, stress reduction, and cognitive function [3, 4]. While these findings are encouraging, limitations such as small sample size and cross‐sectional design limit their generalizability. Although we used selective statistical models (PSM) to reduce selection bias, unmeasured confounders remain. Further longitudinal studies with larger and more diverse populations are needed to confirm and extend these initial observations. Finally, the Satoyama participants were from Hadano City and the comparison group from Kashiwa City. Although both are in the Kanto region, unmeasured regional or environmental differences, such as topography, walkability, and local activity culture, may have influenced the results. Because of the small sample size, we could not adjust for area‐level factors; therefore, residual confounding cannot be ruled out.

In conclusion, this pilot study provides preliminary insights into the potential benefits of nature conservation activities in Satoyama settings for older adults. While our findings suggest possible associations with faster walking speed, better social networks, and lower frailty prevalence, the small sample size and cross‐sectional design limit the generalizability and causal interpretation of the results. Future research with larger and more diverse samples and longitudinal designs is needed to validate and expand upon these initial observations.

## Author Contributions

W.L. designed the study, collected and analyzed the data, and drafted the manuscript. T.T. contributed to study design and critically reviewed the manuscript. M.K. coordinated participant recruitment and data collection. T.K., T.H., and K.K. assisted in field data collection. K.N. and Y.Y. supervised the study and provided editorial support. K.F. provided critical feedback and oversight. K.I. supervised the entire study, secured funding, and contributed to writing and editing. All authors have read and approved the final version of the manuscript.

## Funding

This work was supported by AEON Environmental Foundation.

## Ethics Statement

This study was carried out in accordance with the principles of the Declaration of Helsinki and the Japanese Ethical Guidelines for Medical and Biological Research Involving Human Subjects.

## Supporting information


**Figure S1:** Photographs of Satoyama landscapes.
**Table S1:** Associations between Satoyama activities with older adults' physical, oral function, sociality and frailty by Univariate analysis.
**Table S2:** Associations between Satoyama activities with frailty status by Binary logistic regression.

## Data Availability

The data that support the findings of this study are available on request from the corresponding author. The data are not publicly available due to privacy or ethical restrictions.
