# Supplementary material for: Association of Nature‐Based (Satoyama) Activities and Multiple Health Outcomes Among Older Adults: A Pilot Study
Source: Geriatr Gerontol Int. 2026 Jan 20;26(1):e70358. doi: 10.1111/ggi.70358 (PMC12820443; doi:10.1111/ggi.70358)
Supplement: Supplementary file 1 — Figure S1: Photographs of Satoyama landscapes. Table S1: Associations between Satoyama activities with older adults' physical, oral function, sociality and frailty by Univariate analysis. Table S2: Associations between Satoyama activities with frailty status by Binary logistic regression. [file GGI-26-0-s001.docx]

**Figure S1, Photographs of Satoyama landscapes**

| **Table S1.** Associations between Satoyama activities with older adults’ physical, oral function, sociality and frailty by Univariate analysis. | | | |
| --- | --- | --- | --- |
| **Outcome** | β | 95%CI for β | ***P*** |
| **Physical function** |  |  |  |
| Grip Strength, kg | 0.01 | (-0.01-0.031) | ***.321*** |
| Gait Speed, m/s | 0.83 | (0.06-1.60) | ***.036*** |
| Timed Up and Go test, s | -0.09 | (-0.32-0.15) | ***.464*** |
| ASMI, kg/m^2^ | 0.10 | (-0.05-0.24) | ***.187*** |
| **Oral function** |  |  |  |
| Articulatory oral motor skill, “ka”, times/s | -0.16 | (-0.36-0.05) | ***.130*** |
| GOHAI | 0.10 | (-0.02-0.04) | ***.482*** |
| **Sociality** |  |  |  |
| Social network, lsns6 | 0.03 | (0.00-0.06) | ***.046*** |
| Social support | 0.13 | (-0.08-0.35) | ***.220*** |
| **Frailty score** | -0.17 | (-0.35-0.00) | ***.051*** |
| Abbreviations*:* ASMI, appendicular skeletal muscle mass index; GOHAI, General Oral Health Assessment lndex. Frailty Score: Assessed using the Cardiovascular Health Study criteria, the score ranges from 0 to 5. | | | |

| **Table S2.** Associations between Satoyama activities with frailty status by Binary logistic regression. | | | |
| --- | --- | --- | --- |
|  | Frailty, n (%) | 95%CI | ***P*** |
| No | 20 (71.4%) | 1.00 (reference) | ***-*** |
| Yes | 5 (35.7%) | 0.24 (0.06~0.87) | ***.030*** |
| Abbreviations: OR, odds ratio; CI, confidence interval; Bold typeface indicates statistical significance *(P<0.05)*. | | | |
